# Morphological and Molecular Characterization of *Coslenchus paramaritus* n. sp. and *C. cancellatus* (Cobb, 1925) Siddiqi, 1978 (Nematoda: Tylenchidae) from Iran

**DOI:** 10.21307/jofnem-2019-059

**Published:** 2019-09-17

**Authors:** Manouchehr Hosseinvand, Ali Eskandari, Reza Ghaderi

**Affiliations:** 1 Department of Plant Protection, Faculty of Agriculture, University of Zanjan, 45371-38791, Zanjan, Iran; 2 Department of Plant Protection, School of Agriculture, Shiraz University, 71441-65186, Shiraz, Iran

**Keywords:** Dezful, Khuzestan, new species, 28S rRNA, taxonomy, Tylenchomorpha

## Abstract

*Coslenchus paramaritus* n. sp. and *C. cancellatus* were identified based on morphological, morphometric, and molecular characteristics. The new species, *C. paramaritus* n. sp., is characterized by its cuticle with 18 longitudinal ridges excluding lateral lines, wide cuticular annuli (2.6–3.0 µm), a long tail (94–128 µm), the presence of males and the absence of sexual dimorphism in head shape. Molecular phylogenetic studies of the new species using D2–D3 expansion segments of 28S rRNA revealed that the new species formed a sister clade with three unknown populations of *Coslenchus* in Bayesian inference (BI) phylogeny, while *C. cancellatus* formed a sister clade with other *Coslenchus* species including *C. oligogyrus*, *C. franklinae*, *C. costatus*, *C. turkeyensis* and three unknown populations. *C. cancellatus* is recovered from Iran for the first time.

The genus *Coslenchus* was originally proposed by Siddiqi (1978) to consider of four species in the family Tylenchidae Örley, 1880 with the following main diagnostic characters: “body cuticle coarsely annulated, the presence of longitudinal ridges, labial plate dumbbell-shaped, with round, pore-like amphid apertures not extending on lateral sides of cephalic region, vulva with thick lips and lateral membranes in females, and cloacal lips forming a short tube in males”.

The type species of the genus, *C. costatus* (de Man, 1921) (Siddiqi, 1978) was described on the basis of specimens obtained from moist soil associated with plants on River Mark bank in a suburb of Breda, the Netherlands. This genus currently includes 38 valid species (Geraert, 2008), with 15 species of *Coslenchus* reported from Iran (Karegar, 2018). An undescribed species and a new record for Iranian nematode fauna, recovered from natural habitats, during a survey in Dezful, Khuzestan province, are described.

## Materials and methods

### Sampling, processing and morphological characterization

Soil samples were collected from the rhizosphere of different plants and localities in Iran. Nematodes were extracted by the tray method (Whitehead and Hemming, 1965), killed and fixed by hot FPG (4: 1: 1, formaldehyde: propionic acid: glycerol) and processed to anhydrous glycerol (de Grisse, 1969). Nematodes were mounted in glycerol on permanent slides using paraffin wax and studied using a light microscope, equipped with a Dino-eye microscope eye-piece camera in conjunction with its Dino Capture version 2.0 software. Specimens were identified at species level using available identification keys (Geraert, 2008).

### DNA extraction, PCR and sequencing

Nematode DNA was extracted from single individuals and stored at −20°C until used as PCR template. DNA extraction was performed using the protocols described by Tanha Maafi et al. (2003). The D2–D3 expansion fragments of 28S rRNA were amplified using the forward D2A (5'-ACAAGTACCGTGAGGGAAAGT-3') and reverse D3B (5'-TCGGAAGGAACCAGCTACTA-3') primers (Subbotin et al., 2006). The 30 μl PCR contained 15 μl 2× Taq DNA polymerase mix (Ampliqon, Denmark), 1 μl (10 pmol μl^−1^) each of forward and reverse primers, 2 μl of DNA template and 11 μl deionized water. PCR cycling conditions were as follows: denaturation at 95 °C for 4 min, then 33 cycles of denaturation at 94 °C for 30 sec, annealing at 57°C for 30 sec, and extension at 72°C for 90 sec, with a final extension was performed at 72°C for 10 min. The quality of PCR was checked by electrophoresis of 4 μl of the PCR reaction in 1% agarose gel containing ethidium bromide and products were visualized and photographed under UV light. The length and concentration of each PCR product were measured by comparison with a low DNA mass ladder (Invitrogen, Carlsbad, CA). The PCR product was purified and sequenced directly for both strands using the same primers with an ABI 3730XL sequencer (Bioneer, Seoul, South Korea). The newly obtained sequences were submitted to GenBank database under accession numbers MK542004 for *C. paramaritus* n. sp. and MK542005 for *C. cancellatus*.

### Phylogenetic analyses

For phylogenetic relationships, analyses were based on D2–D3 expansion fragments of the 28S rRNA gene sequences. The newly obtained sequences were edited and aligned with another segments of 28S rRNA gene sequences available in GenBank using MUSCLE alignment tool implemented in the MEGA7 (Kumar et al., 2016). The best-fit model of nucleotide substitution used for the phylogenetic analysis was statistically selected using jModelTest 2.1.10 (Darriba et al., 2012) and phylogenetic tree was generated with the Bayesian inference method using MrBayes 3.2.6 (Huelsenbeck and Ronquist, 2001; Ronquist et al., 2012). The analysis under GTR+I+G model was initiated with a random starting tree and run with the Markov Chain Monte Carlo (MCMC) for 1 × 106 generations. The tree was visualized and saved with FigTree 1.4.3 (Rambaut, 2014) and edited with Adobe^®^ Acrobat^®^ XI Pro 11.0.1. A sequence of *Ditylenchus dipsaci* (FJ707361) was chosen as outgroup for 28S tree.

## Results

### Systematics

**Figure 1:**
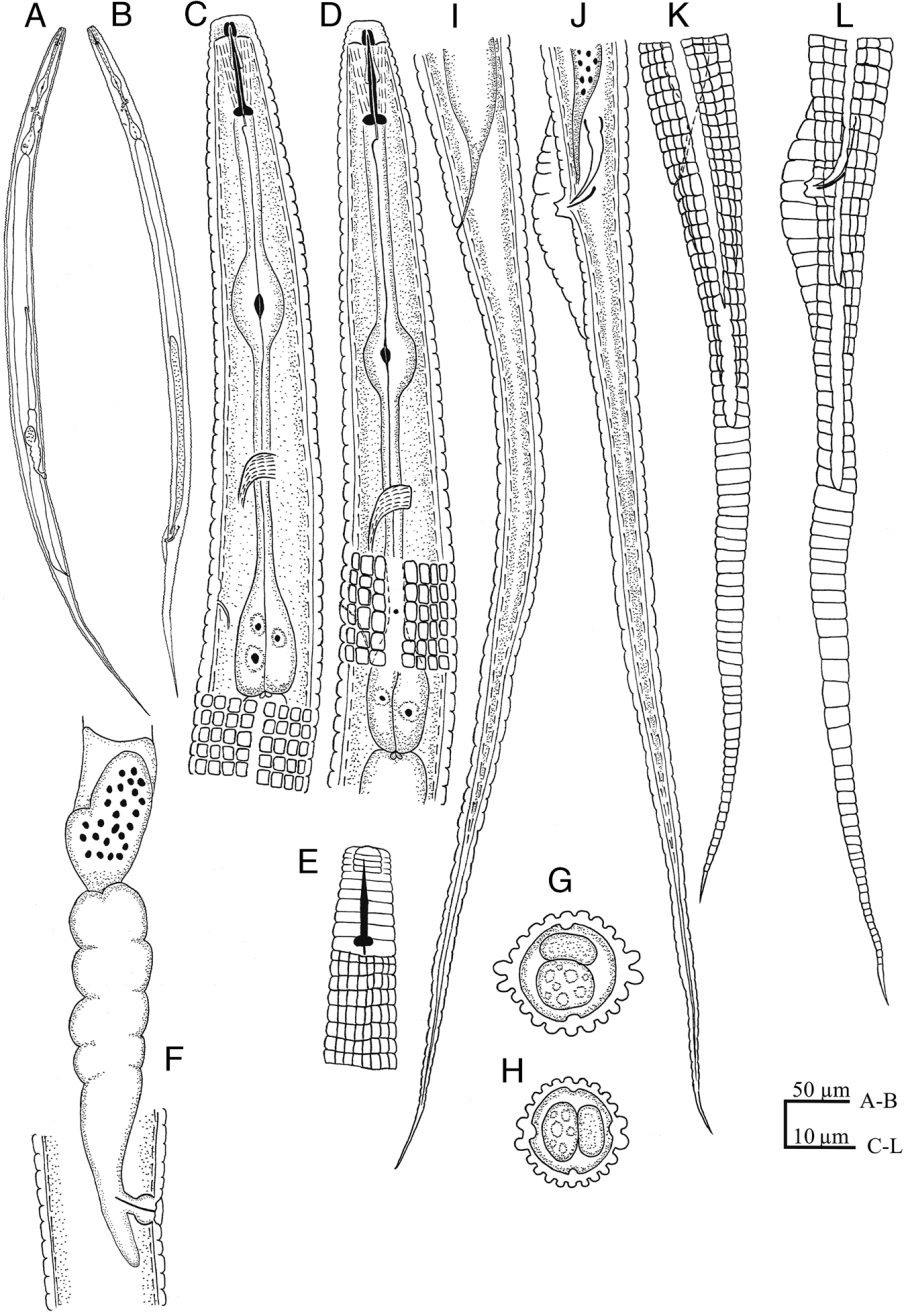
Figure 1: *Coslenchus paramaritus* n. sp. (line drawing). (A, B) Entire body in female and male. (C, D) Anterior portion. (E) Anterior end in dorso-ventral view. (I, K) Female tail. (J, L) Male tail. (F) Reproductive system. (G, H) Cross section at mid-body.

**Figure 2:**
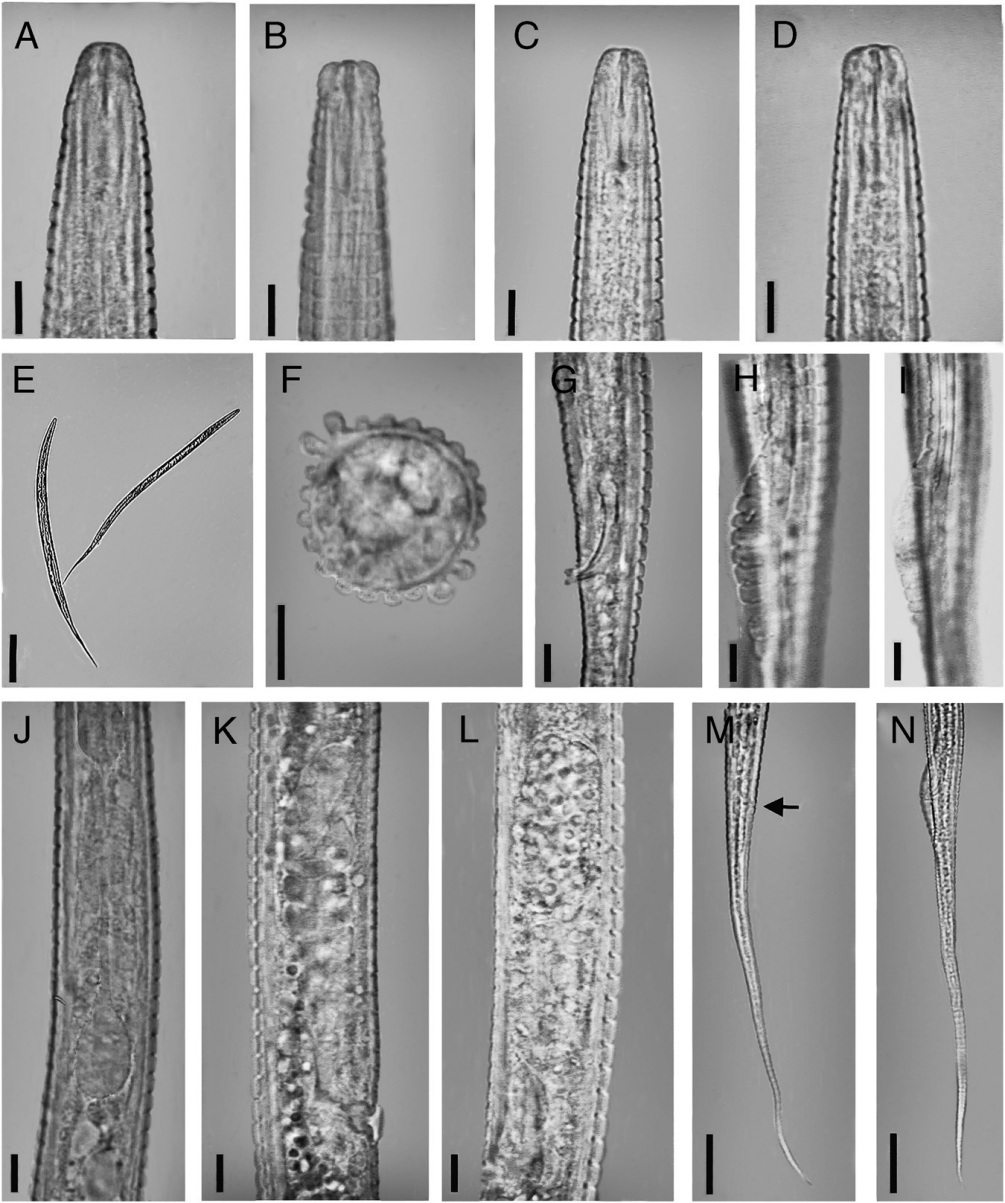
Figure 2: *Coslenchus paramaritus* n. sp. (light photograph). (A) Female Anterior end (Lateral view). (B) Female Anterior end (Dorso-ventral view). (C) Male anterior end (Lateral view). (D) Male anterior end (Dorso-ventral view). (E) Entire body. (F) Cross section at mid-body. (G–I) Spicules and Bursa. (J) End portion of esophagus. (K, L) Female reproductive system. (M) Female tail. (N) Male tail. (A–D, F–L = 5 µm, E = 100 µm, M–N = 20 µm).

*Coslenchus paramaritus* n. sp.

(Figs. 1 & 2; Table 1).

**Table 1.** Morphometric characters of *Coslenchus paramaritus* n. sp. and *C. cancellatus* from Iran (measurements are in µm).

	*C. paramaritus* n. sp.					*C. cancellatus*	
Character\Species	Holotype	Females		Males		Females	
n	–	10	CV	8	CV	12	CV
L	519	537 ± 22.3 (508–572)	4.2	529 ± 31.4 (474–571)	5.9	446 ± 29.9 (388–481)	6.7
a	26.6	27.3 ± 3 (22.5–33.6)	11.1	36 ± 1.6 (33.7–38.2)	4.6	25.6 ± 1.8 (23.2–28.1)	7.3
b	5.9	5.4 ± 0.3 (5.1–6.1)	5.9	14.8 ± 0.4 (14–15.3)	2.9	4.7 ± 0.4 (3.4–5.1)	10.6
c	4.5	4.7 ± 0.4 (4.3–5.7)	9.6	4.6 ± 0.1 (4.3–4.9)	4.0	5.8 ± 0.5 (4.9–6.5)	9.1
c′	11.9	10.7 ± 1.2 (7.8–11.8)	11.5	11.3 ± 0.9 (10.1–13)	8.6	7.5 ± 0.7 (6.5–8.4)	9.6
V or T	62.6	63.8 ± 2.3 (61.8–69.6)	3.7	34 ± 4.1 (28.6–39.4)	12.1	66.4 ± 1.5 (64.6–69.9)	2.3
V'	80.4	80.5 ± 1.9 (76.7–83.2)	2.4	–	–	80.2 ± 1.8 (77–82.9)	2.3
Stylet	13.5	12.8 ± 0.6 (12–13.9)	5.2	12.4 ± 0.2 (12–12.9)	2.3	10.9 ± 0.3 (10.5–11.4)	2.8
m (conus/stylet %)	45.3	46.1 ± 1.8 (43.2–48.3)	3.9	47.8 ± 1.3 (45.8–49.5)	2.7	44.8 ± 2 (42.2–48.5)	4.7
Pharynx	90	97.9 ± 5.8 (85–107)	6.0	97.1 ± 4.4 (92–103)	4.6	95.4 ± 9 (85–112)	9.5
Median bulb	44.5	44.6 ± 2.1 (41–47)	4.9	44.6 ± 2.2 (42–49)	5.1	41.9 ± 3.8 (38–50)	9.3
MB	47	45.6 ± 2.5 (42.4–51.7)	5.6	45.9 ± 1.1 (44.5–47.5)	2.4	43.9 ± 1.4 (41.8–46.6)	3.2
Excretory pore	79	81 ± 3.3 (76–86)	4.2	79.9 ± 3 (74–83)	3.8	75.6 ± 12 (59–103)	15.9
Head-vulva	325	340 ± 17.6 (314–370)	5.2	–	–	296 ± 18.2 (257–319)	6.2
Head-anus	404	423 ± 22.7 (394–459)	5.4	414 ± 24.1 (373–442)	5.8	369 ± 28 (310–401)	7.6
Vulva–anus	89	82.6 ± 10.5 (71–104)	12.8	–	–	73 ± 11.5 (53–92)	15.8
Body width	19	19.6 ± 1.6 (17–22)	8.6	14.7 ± 1.1 (12.5–15.8)	7.5	17.4 ± 2.1 (14.2–20.7)	12.5
Vulval body width	16	17.1 ± 1 (15.5–19)	6.3	–	–	15.2 ± 1.5 (13–17)	10.3
Anal body width	9.7	10.6 ± 0.6 (9.7–12)	5.8	10.1 ± 0.2 (10–10.6)	2.3	10.2 ± 0.6 (9–11)	6.5
Tail/Vulva–Anus	1.3	1.4 ± 0.2 (0.9–1.8)	17.4	–	–	1 ± 0.1 (0.7–1.4)	17.6
Annulus width	2.8	2.8 ± 0.1 (2.6–3)	5.1	2.8 ± 0.2 (2.4–3)	7.2	2.9 ± 0.2 (2.4–3.3)	7.6
Tail length	115	114 ± 9.9 (94–128)	8.7	115 ± 8.9 (101–130)	7.7	76.8 ± 6.9 (65–87)	9.0
R	216	222.4 ± 10 (210–240)	4.5	214.6 ± 10.7 (201–233)	5.0	188.5 ± 13.8 (170–210)	7.3
Rv	85	86 ± 5.8 (76–96)	6.7	–	–	65.6 ± 7.5 (58–83)	11.5
Ran	54	53.2 ± 6.4 (41–59)	12.2	53.8 ± 4 (48–60)	7.5	31.7 ± 2.8 (28–36)	9.0
Rvan	30	32.8 ± 4.5 (26–40)	13.9	–	–	33.9 ± 6.3 (27–47)	18.7
Gubernaculum	–	–	–	5.1 ± 0.3 (4.5–5.5)	6.7	–	–
Spicules	–	–	–	14.3 ± 0.8 (13–15.5)	6.3	–	–

#### Description

##### Female

Body almost straight to slightly ventrally curved. Body annuli distinct, 2.6 to 3.0 µm wide at mid-body, and 2.3 to 2.7 µm at the Esophageal region. Lateral field protruded, longitudinal incisures begin 5 to 8 annuli posterior to head and continue until 15 to 22 annuli to anus, with two prominence ridges and four incisures, 5.3 to 7.5 µm wide, covering 26 to 40% of body. Cuticle with further 18 longitudinal ridges excluding lateral lines. Head continuous to the body contour or slightly offset by a depression, 2.8 to 4.0 µm high and 5.8 to 6.9 µm wide, narrower than the rest of body. Head with distinct transverse striae separating four narrow annuli, cephalic framework weakly developed. Amphidial apertures indistinct. Stylet short and delicate, with distinct knobs 2.0 to 3.2 µm wide, conus less than half of the total stylet length, 5.2 to 6.6 µm long; dorsal gland orifice 1.0 to 2.0 µm behind stylet knobs. Esophageal median bulb oval, 9.8 to 12.3 µm in length and 5.9 to 7.7 µm in width, filling 39 to 54% of the body diameter with small valve apparatus. Terminal bulb pear-shaped, 14 to 19 µm long and 7.7 to 9.5 µm wide. Excretory pore 76 to 86 µm from anterior end, or anterior to terminal bulb, at level or one annulus posterior to hemizonid; Nerve ring encircling middle of isthmus, 62 to 73 µm from anterior end. Deirids located at the same level, 76 to 87 µm from anterior end. Esophageal-intestinal valve distinct. Rectum small, curved. Anus usually indistinct. Female reproductive system monodelphic, anteriorly directed; vulva with lateral flaps about two annuli long. Vagina with thick walls, slightly bent anteriorly; post-vulval uterine sac short, 8 to 12 µm, about half of the vulval body width (45 to 60%). Spermatheca offset, usually bilobed, filled with globular to slightly oval sperm, about 1 µm in diameter. Tail elongated, with sharply pointed to filiform terminus.

##### Male

Body straight to ventrally curved. Cuticle annuli 2.4 to 3.0 µm apart at mid-body and 2.3 to 3.0 µm at the Esophageal region. Lateral field 4.5 to 5.0 µm wide, occupying 30 to 36% of the body diameter, with two prominent separate ridges, hence four distinct incisures can be observed on mostly body length, without areolation. The cephalic region truncated, continuous or slightly set off from body with four annuli, 6.1 to 6.8 µm wide and 3.6 to 4.1 µm high. Cephalic framework not refractive. Stylet knobs well developed and globular, 2.1 to 2.5 µm in diameter. Esophageal median bulb oval, (5.8–6.5) × (9.8–12.0) µm. Bursa limited to the cloacal region, with crenate margins. Spicules slightly curved ventrally, with pointed tip. Gubernaculum simple, about one-third of spicule length. Tail elongated ending to a pointed to filiform terminus.

##### Diagnosis and relationships

The new species, *Coslenchus paramaritus* n. sp., can be characterized by having wide annuli, a long tail, the presence of males and the absence of sexual dimorphism in head shape of females and males. *Coslenchus paramaritus* n. sp. most closely resembles *C. maritus* Andrássy, 1991, but differs from it by wider cuticular annuli (2.6–3.0 µm vs 1.5–2.3 µm), longer tail (94–128 µm vs 83–91 µm), and the absence of sexual dimorphism in head shape (vs presence, male head is the widest in its middle). However, in general characteristics and number of longitudinal ridges, *C. paramaritus* n. sp. resembles certain other species namely *C*. *lateralis* (Andrássy, 1982), *C*. *major* Gagarin, 2004, *C*. *franklinae* (Siddiqi, 1981), *C*. *leiocephalus* (Brzeski, 1998), *C. japonicus* (Mizukubo and Minagawa, 1984), and *C*. *areolatus* (Egunjobi, 1967) (Siddiqi, 1978).

The new species differs from *C*. *lateralis*, by longer stylet (12–14 vs 11–12 µm), number of longitudinal ridges in the lateral field region (6 vs 4), and presence of male individuals (vs absence). From *C. major*, it can be distinguished by smaller body, stylet and tail (508–572 vs 780-990 µm, 12–14 vs 17–18 µm and 94–128 vs 133-168 µm, respectively), and spermatheca shape (elongated-bilobed vs elongated-rounded, 28–39 µm long). From *C. franklinae*, our population can be differentiated by longer stylet (12–14 vs 9–12 µm), number of annuli on the cephalic region (4 vs 2-3), and the presence of male individuals (vs absence). From *C*. *leiocephalus*, our population can be distinguished by a longer stylet (12-14 vs 10-12 µm), the number of head annuli (4 annuli vs smooth head), presence of a post-vulval uterine sac (vs absence) and presence of males (vs absence). The new species differs from *C. japonicus* by higher values for body length, pharynx length, tail length, cuticular annuli width, the number of head annuli and spicules length (508-572 vs 430-450 µm, 85-107 vs 77-89 µm, 94-128 vs 73-99 µm, 2.6-3.0 vs 1.9-2.5 µm, 4 vs 3 and 13-15.5 vs 11-14 µm, respectively), as well as presence of post-vulval uterine sac (vs absence). *C. paramaritus* n. sp. differs from *C. areolatus* in the presence of male individuals (vs absence), having a longer stylet (12-14 vs 10-12 µm), annuli number of cephalic region (4 vs 3) and in the shape of tail (elongated, terminus sharply pointed to filiform vs thick tail often covered by small scale-like annuli, terminus always finely rounded).

##### Type habitat and locality

Soil around of wild fig (*Ficus carica* subsp. *rupestris*) in Dezful, Khuzestan Province, southwestern Iran (GPS coordinates: 48°49'30″N, 32°46'24″E).

##### Type material

Holotype, 10 paratype females and 7 males were deposited in the collection of the Department of Plant Protection, College of Agriculture, University of Zanjan, Zanjan, Iran.

##### Etymology

The species epithet refers to the proximity of the new species with the other known species, *C. maritus*.

**Figure 3:**
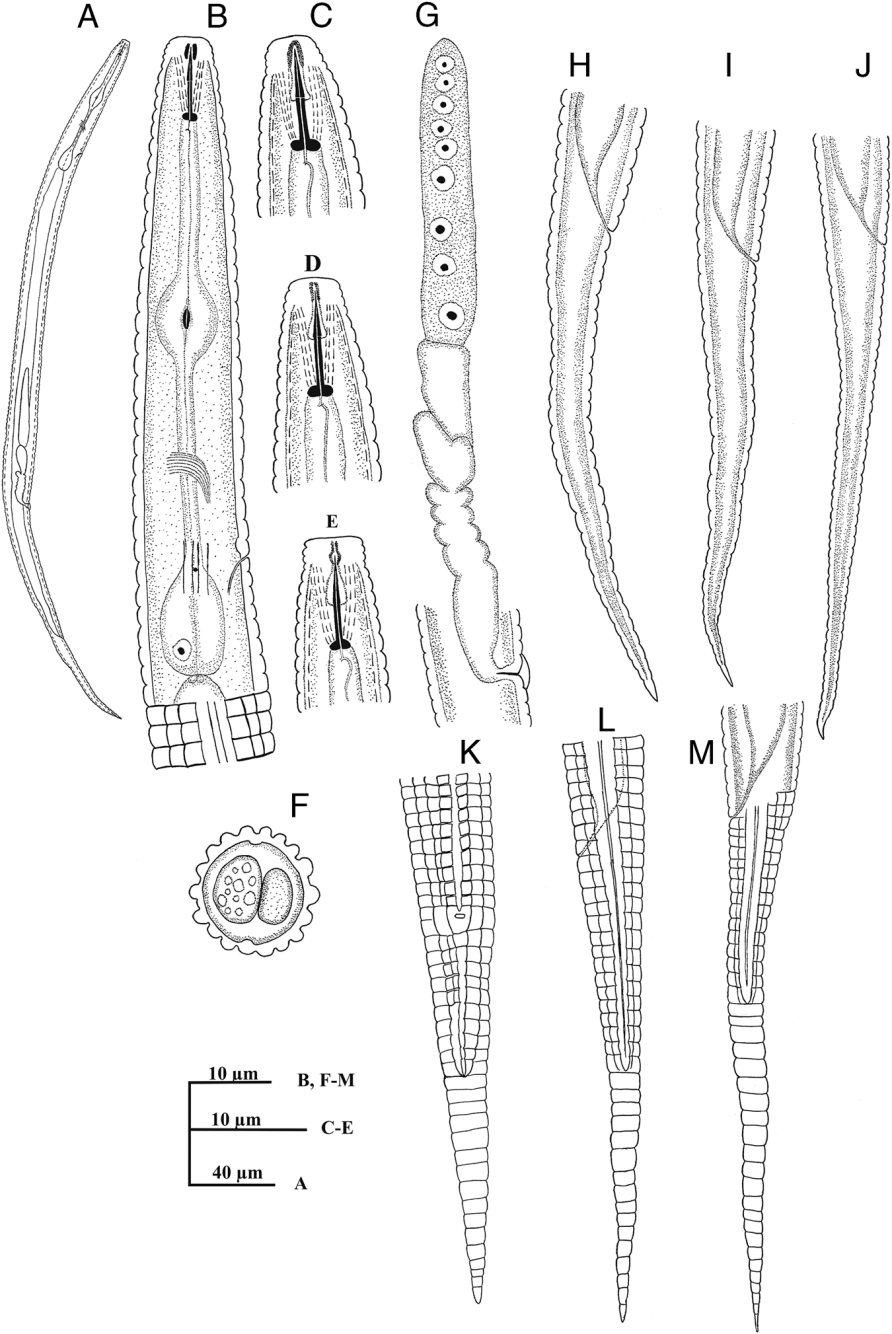
Figure 3: *Coslenchus cancellatus* (Cobb, 1925) Siddiqi, 1978 (line drawing). (A) Entire body in female. (B) Anterior portion. (C, D) Anterior end in lateral view. (E) Anterior end in dorso-ventral view. (F) Cross section at mid-body. (G) Reproductive system. (H–M) Female tail.

**Figure 4:**
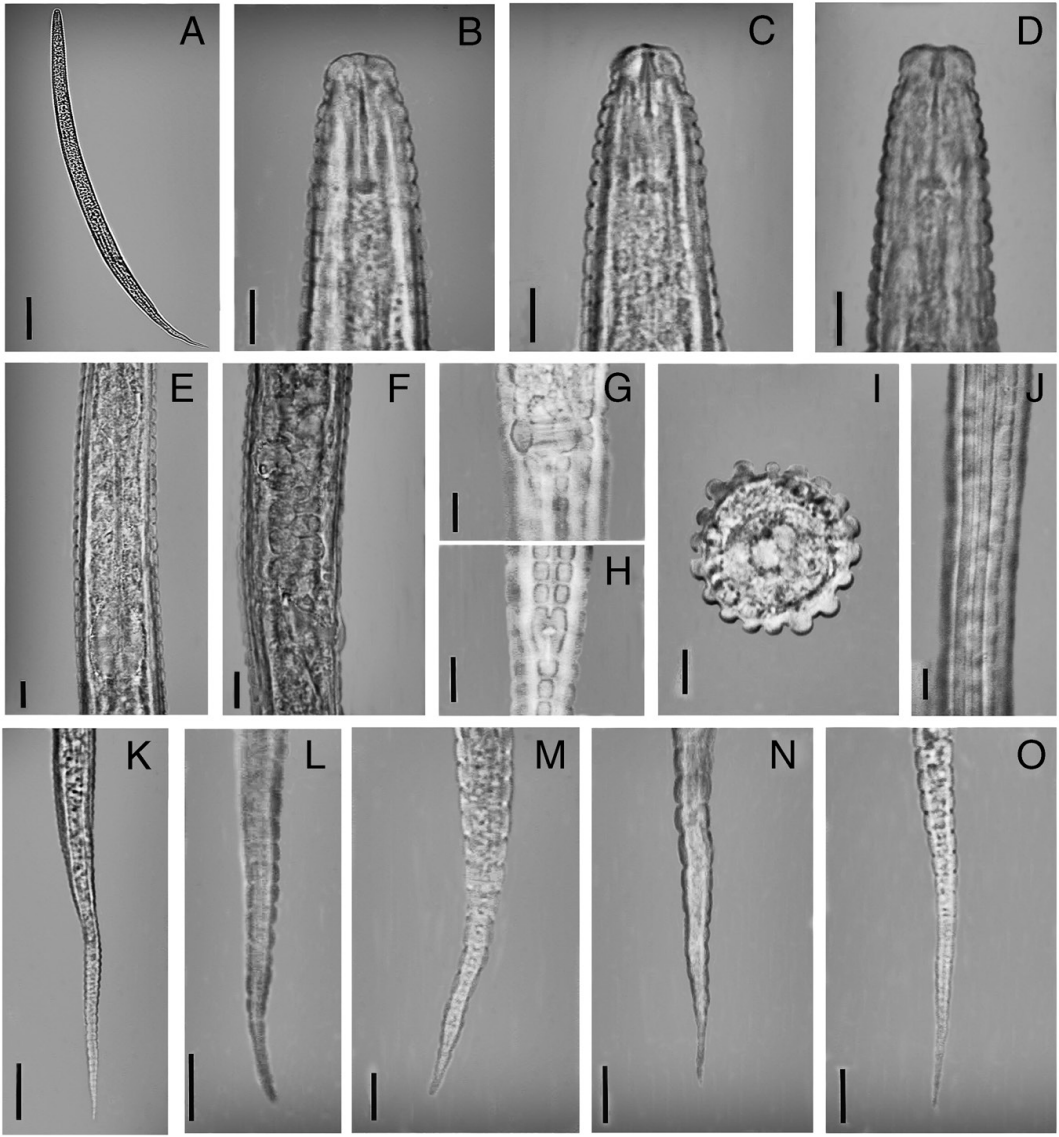
Figure 4: *Coslenchus cancellatus* (Cobb, 1925) Siddiqi, 1978, female (light photograph). (A) Entire body. (B, C) Anterior end in lateral view. (D) Anterior end in dorso-ventral view. (E) Posterior of esophagus. (F) Reproductive system. (G) Front view of vulva. (H) Front view of anus. (J) Lateral field. (K) tail. (L–O) Posterior of tail. (A = 20 µm, B–G, L–O = 5 µm, K = 50 µm.)

*Coslenchus cancellatus* (Cobb, 1925) Siddiqi, 1978.

(Figs. 3 & 4; Table 1).

#### Description

##### Female

Body almost straight to slightly ventrally curved. Body annuli pronounce and wide, 2.4 to 3.3 µm at mid-body, and 2.3 to 2.9 µm at the posterior region of esophagus. Lateral field protruded, with two prominence ridges, 4.0 to 7.5 µm wide, occupying 28 to 39% of the body diameter. Head slightly offset by a depression, 2.5 to 3.3 µm high and 6.3 to 7.1 µm wide, narrower than its adjacent body, cephalic framework weakly developed. Amphidial apertures indistinct. Stylet short and delicate, with distinct knobs 2.2 to 2.7 µm wide; conus less than half of stylet length, 4.6 to 5.3 µm long, dorsal gland orifice 1.0 to 1.8 µm behind stylet knobs. Esophagus median bulb oval, 7.8 to 10.3 µm wide, filling 52 to 66% of the corresponding body diameter. Posterior bulb pear-shaped, 15 to 20 µm long and 8 to10 µm wide. Excretory pore from anterior end 59 to 103 µm, i.e. at the level of terminal bulb, at the same level with hemizonid or posterior to it; deirids at the same level or slightly posterior. Nerve ring encircling middle of isthmus, 55 to 78 µm from anterior end. Deirids at the level of excretory pore, 68 to 110 µm from anterior end. Esophageal-intestinal valve distinct. Rectum small, curved. Anus usually indistinct. Female reproductive system monodelphic-prodelphic. Lateral vulval flaps about two annuli or 4.2 to 6.8 µm long; vagina bent anteriad with thick walls, post-vulval uterine sac absent. Spermatheca offset, devoid of sperm. Tail conical with finely rounded to pointed terminus.

##### Male

Not found.

##### Remarks

This species was first described by Cobb (1925) from USA as *Tylenchus cancellatus* (Cobb, 1925), then Siddiqi (1978) transferred it to the genus *Coslenchus*. This species is most similar to *C*. *costatus* (de Man, 1921) (Siddiqi, 1978) and *C. pycnocephalus* (Siddiqi, 1981) in having 14 longitudinal ridges. It differs from *C. costatus* in the shape of posterior part of tail (pointed to minutely rounded vs spicate or filiform) and width of cuticular annuli at mid-body (about 4.0 vs 2.1-3.6 µm) and lesser number of tail annuli (28-36 vs more than 50). It differs from *C*. *pycnocephalus* in the head structure (smooth or having only one head annulus *vs* annulated with four or five distinct annuli). In general appearance *C*. *cancellatus* is also comparable with *C*. *oligogyrus* (Brzeski, 1987); but can be distinguished form it by different number of longitudinal ridges and head annuli (14 vs 10-12 and usually 5 vs 3, respectively) and a different ratio of tail to vulva–anus distance (1.2-1.6 vs 0.8-1.2).

Our population was recovered from the rhizosphere of Alpine Milkvetch (*Astragalus alpinus* L.) in Dezful, Khuzestan Province, southwestern Iran. Voucher specimens including 12 females were deposited in the collection of the Department of Plant Protection, College of Agriculture, University of Zanjan, Zanjan, Iran.

#### Molecular phylogenetic status

**Figure 5:**
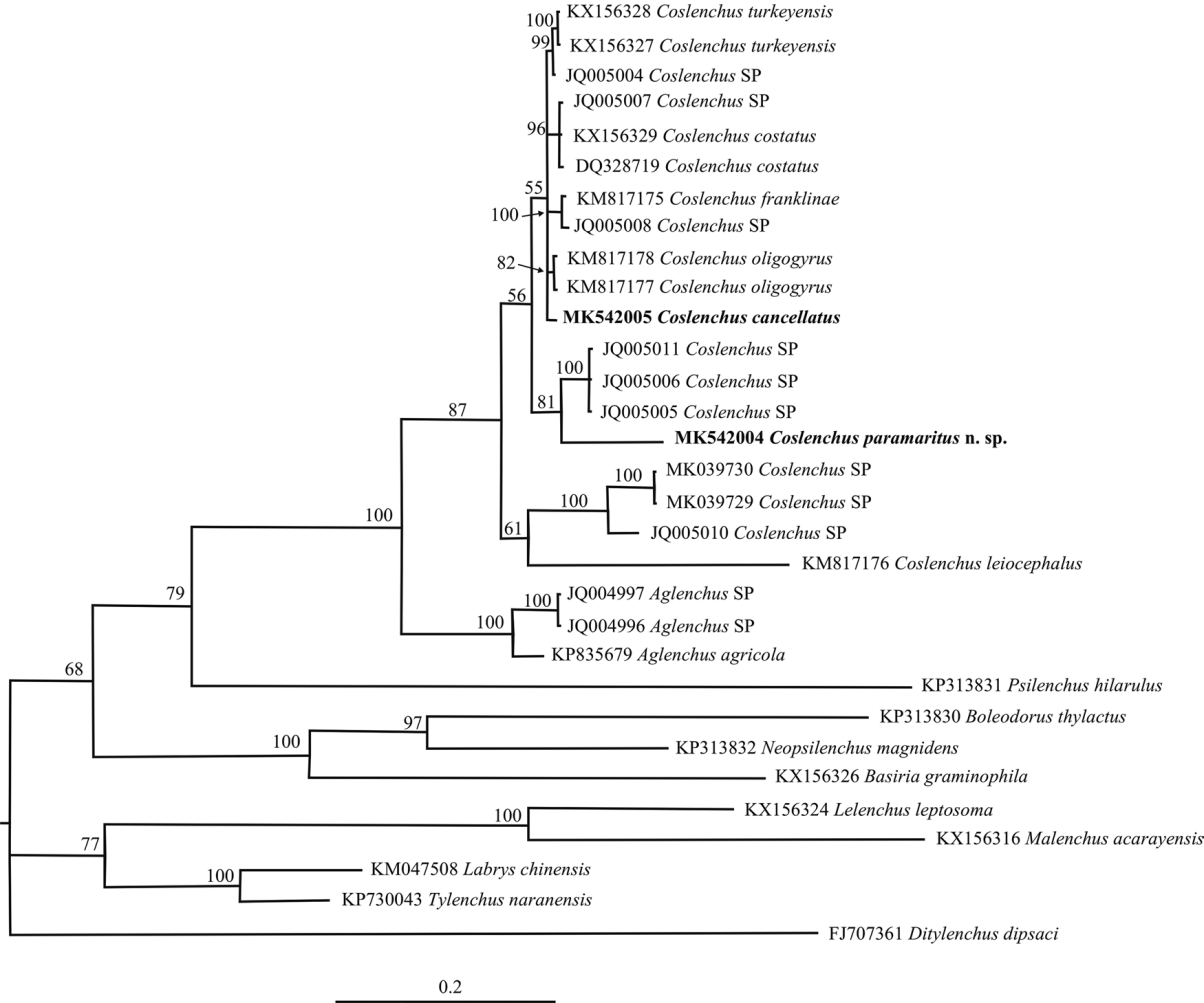
Figure 5: The 50% majority rule consensus trees from Bayesian analysis generated from the D2–D3 expansion fragments of 28 S rRNA gene dataset under GTR+I+G model. Posterior probabilities for BI analysis more than 50% are given for appropriate clades. New sequences are indicated in bold.

The amplification of D2-D3 expansion fragments of 28S rRNA gene sequences of *C. paramaritus* n. sp. and *C. cancellatus* yielded a single fragment of 736 (after editing 635) and 751 (after editing 642) bp, respectively. The D2-D3 expansion fragment of 28S rRNA alignment contained 30 ingroups and *Ditylenchus dipsaci* as outgroup taxon and was 720 bp in length after removing ambiguously aligned regions. The 50% majority rule consensus phylogenetic tree generated from the D2–D3 alignment by BI analysis under GTR+I+G model is presented in Figure 5. Sequences of *C*. *paramaritus* n. sp. and *C*. *cancellatus* matched well with those of other known species of *Coslenchus* and *Aglenchus* deposited in GenBank. *C. paramaritus* (MK542004) and *C*. *cancellatus* (MK542005) formed a basal clade clustering (PP = 81%) with two isolates of an unknown species of *Coslenchus* (JQ005005 and JQ005006), but separated from the sequences of other *Coslenchus* species.

## Discussion

In our phylogeny tree, the new species formed a sister clade with three unknown populations of *Coslenchus* (JQ005005, JQ005006, and JQ005011). The other species, *C. cancellatus* formed a sister clade with other *Coslenchus* species including *C. oligogyrus*, *C. franklinae*, *C. costatus*, *C. turkeyensis* and three unknown populations. Our current knowledge of the taxonomy of the genus *Coslenchus* mostly comes from classic morphological and morphometric data. Current molecular data in GenBank are only available for a few species; however, some of them are not linked to published descriptions and therefore the morphological characters of those isolates are unknown. The inclusion of new or existing representatives of *Coslenchus* provides an opportunity to obtain a better insight into the intra- and inter-generic structure of the genus within other members of the family Tylenchidae.
